# *In vitro* Anti-parasitic Activity of *Pelargonium X. asperum* Essential Oil Against *Toxoplasma gondii*

**DOI:** 10.3389/fcell.2021.616340

**Published:** 2021-02-18

**Authors:** Si-Yang Huang, Na Yao, Jia-Kang He, Ming Pan, Zhao-Feng Hou, Yi-Min Fan, Aifang Du, Jian-Ping Tao

**Affiliations:** ^1^Jiangsu Key Laboratory of Zoonosis, Jiangsu Co-innovation Center for Prevention and Control of Important Animal Infectious Diseases and Zoonosis, Institute of Comparative Medicine, College of Veterinary Medicine, Yangzhou University, Yangzhou, China; ^2^Joint International Research Laboratory of Agriculture and Agri-Product Safety, The Ministry of Education of China, Yangzhou University, Yangzhou, China; ^3^College of Animal Science and Technology, Guangxi University, Nanning, China; ^4^Zhejiang Provincial Key Laboratory of Preventive Veterinary Medicine, College of Animal Sciences, Institute of Preventive Veterinary Medicine, Zhejiang University, Hangzhou, China

**Keywords:** *Toxoplasma gondii*, natural extraction products, drug, development, *Pelargonium X. Asperum* EO

## Abstract

Toxoplasmosis is a global zoonotic disease, and one-third of the human population is chronically infected by *Toxoplasma gondii*. Due to the limited effectiveness and prominent side effects of the existing drugs, there is a dire need for the discovery of new therapeutic options in the treatment of toxoplasmosis. In this study, five essential oils (EO) were screened for their anti-parasitic activity against *T. gondii*. The cytotoxicity of essential oils was evaluated using the MTT assay on human foreskin fibroblast cells. The CC_50_ values of *Eucalyptus globulus* EO, *Cupressus sempervirens* EO, *Citrus aurantifolia* EO, *Melaleuca alternifolia* EO, and *Pelargonium X. asperum (Pa)* EO were found to be 22.74, 7.25, 15.01, 6.26, and 4.77 mg/mL, respectively. Only *Pa*EO exhibited anti-parasitic activity, and inhibited the growth of *T. gondii* in a dose-dependent manner. In addition, treatment with *Pa*EO, was found to reduce the volume of *T. gondii* tachyzoites and make their membrane surfaces rough. These results showed that *Pa*EO was able to inhibit the growth of *T. gondii* by reducing invasion, which may be due to its detrimental effect on the ability of tachyzoites to move. These findings suggest that *Pa*EO could be a potential anti-*T. gondii* drug, which may facilitate the development of new and effective treatments against toxoplasmosis.

## Introduction

*Toxoplasma gondii* is an opportunistic parasite that infects most warm-blooded animals, causing ophthalmopathy, abortion, stillbirth, and choroidal retinitis (Zhang et al., 2016). The parasite is transmitted to humans through the ingestion of raw meat contaminated with tissue cysts, or through food or water contaminated with oocysts. The oocyst’s walls protect the cells from the external environment, making them resistant to the chlorine dioxide and chloramine used to disinfect water (Shapiro et al., 2019). Serious toxoplasmosis outbreaks have occurred in several countries, both in humans and in animals, leading to serious economic and public health problems.

Since the 1950s, a combination of sulfadiazine and pyrimethamine has been the standard therapy for toxoplasmosis (Eyles and Coleman, 1953). Unfortunately, the side effects of this combination are serious, and very often, patients cannot complete the entire course of treatment because of drug intolerance. Spiramycin was initially used in the treatment of *T. gondii* infection in 1958. While this drug does reduce mother-to-child transmission, its inability to cross the placental barrier means that it is unable to reach an infected fetus (Desmonts and Couvreur, 1974). Clindamycin, clarithromycin, and trimethoprim-sulfamethoxazole (TMX-SMX) have also been used in the treatment of toxoplasmosis. However, overuse of these drugs over the years has led to the emergence of drug resistance (Dunay et al., 2018). Therefore, the development of new drugs to control and treat toxoplasmosis in both humans and animals, is the need of the hour.

Natural products are a good alternative to synthetic molecules for the treatment of *T. gondii* infections. Products isolated from natural sources are superior to synthetic molecules in terms of their impact on the environment, source of acquisition, and safety profile (Petrovska, 2012). Through long-term interactions and co-evolution with other plants and species in their habitat, certain plants have evolved to produce a large number of structurally diverse secondary metabolites, which possess a variety of ecological functions that are essential for their survival (Mahizan et al., 2019). Essential oils (EOs) are a mixture of secondary metabolites, composed mainly of terpenes, aldehydes, and esters (Swamy et al., 2016). Most hydrophobic molecules that are small can cross biological barriers and biofilms (Costa et al., 2018); prevent infections, inflammation, and spasms; fight bacteria and viruses (Gucwa et al., 2018); and promote cell metabolism and regeneration. Certain components of EOs possess anti-parasitic activity.

An extract prepared from the leaves of *Eucalyptus globulus* (*Eg*) has been shown to exhibit a significant inhibitory effect on *Aggregatibacter actinomycetemcomitans* and *Porphyromonas gingivalis in vitro* (Bankur et al., 2019). In addition, it can also kill *Aedes mosquito* (Kaura et al., 2019) and inhibit the growth of *Giardia lamblia* cysts (Dehghani-Samani et al., 2019). Two hydrogenated monoterpenes, extracted from *Cupressus sempervirens* (*Cs*) have been shown to possess pesticidal properties against *Sitophilus zeamais* (Langsi et al., 2020). *Citrus aurantifolia* (*Ca*) belonging to *the Rutaceae* family can effectively ward off mosquitoes (Araujo et al., 2016). *Melaleuca alternifolia* EO (*Ma*EO) is a promising candidate for use as a repellent, acaricide, and insecticide (Souza et al., 2016). The Japanese beetle (*Popillia japonica*) is paralyzed by consuming *Pelargonium hortorum*, a plant belonging to the same genus as *Pelargonium X. asperum* (*Pa*) (Ranger et al., 2011). The EOs in these plants exhibit insecticidal, anti-bacterial and anti-fungal properties, among a range of other biological functions.

EOs that were extracted from several plant species have been reported to exhibit activity against *T. gondii.* EO from *Thymus broussonetii Boiss*, has been shown to reduce prugniaud (Pru) cysts (Dahbi et al., 2010). *Bunium persicum* (Boiss) EO has been shown to prevent *T. gondii* infection in mice and significantly delay their time of death (Kareshk et al., 2015). These results suggest that EOs may possess anti-*T. gondii* activity. In order to verify this hypothesis, we screened five EOs from different plants for their anti-*T. gondii* activity.

## Materials and Methods

### Cell Culture and Parasite

*T. gondii* tachyzoites of the RH strain, expressing green fluorescence protein (GFP-RH) were maintained in human foreskin fibroblast (HFF) cells, cultured in Dulbecco’s modified Eagle’s medium (DMEM), supplemented with 100 IU/mL penicillin and 100 μg/mL streptomycin, along with 10% heat-inactivated fetal bovine serum (FBS). The culture was incubated at 37°C, in an atmosphere containing 5% CO_2_. To isolate the tachyzoites, heavily infected cells were scraped and the parasites were released by passing the cells through a 27-gauge needle, 3–5 times. Cell debris was removed by passing the mixture through a 3-μm pore membrane filter (Whatman, Thermo Fisher Scientific, Waltham, MA, United States). Tachyzoites were quantified using a hemocytometer before proceeding to further experiments.

### Essential Oils

The EOs were purchased from the French EO manufacturer, Florihana. These included the EOs of *Pa* (Lot Number: AM020920MG), *Ma* (Lot Number: AM020920MG), and *Eg* (Lot Number: E060420ES), extracted from their leaves; the EO of *Ca* (Lot Number: B180719BR), extracted from the plant zest; and the EO of *Cs* (Lot Number: A010420F), extracted from its branches. All EOs were extracted by steam distillation. The main ingredients and chemical components of each EO are summarized in Supplementary Table 1. All EOs were dissolved in dimethyl sulfoxide (DMSO) in a 1:1 ratio. The solutions were then diluted with DMEM, such that the final concentration of DMSO in the samples used in the experiment was lower than 1.56% v/v.

### Cytotoxicity Assay

The cytotoxicity of all these EOs was evaluated in an HFF cell line, using a CellTiter 96^®^ AQueous One Solution Cell Proliferation Assay (Promega Corp., Madison, WI, United States), according to the manufacturer’s instructions. HFF cells (1 × 10^5^ cells/well) were cultured in 96-well plates at 37°C, in an atmosphere containing 5% CO_2_, for 24 h. The cells were treated with varying concentrations of EOs or sulfamethoxazole (SMZ), and incubated for 24 h. Different concentrations of each EO were added to the wells. A 1.56% solution of DMSO in DMEM was used as the vehicle control. After incubating for 48 h, 20 μL of MTS solution, containing phenazine ethyl sulfate, was added to each well, and incubated for 3 h at 37°C. Absorbance was measured at 490 nm using an iMark^TM^ Microplate Absorbance Reader (BioRad, Hercules, CA, United States). Wells containing cells treated only with DMEM were used as the negative control (Montazeri et al., 2019). The 50% cytotoxic concentrations (CC_50_) were calculated using Graph Pad Prism 8.0. The cytotoxicity experiment was performed in triplicate, using three separate plates.

### Anti-*T. gondii* Activity of EOs Evaluated by a Plaque Assay

One-hundred freshly released tachyzoites were added to HFF monolayers in 6-well plates, in DMEM with 2% FBS. They were incubated at 37°C, in an atmosphere containing 5% CO_2_, for 4 h. Then, the medium containing extracellular parasites was removed, and fresh medium containing various concentrations of EOs, or 1.56% DMSO in DMEM (vehicle control) was added to each well. Uninfected and untreated wells were used as blank controls. After 7 days, HFF cells were washed three times with PBS, fixed with methanol for 10 min, and stained with 0.1% crystal violet for 30 min. After washing three times with phosphate buffered saline (PBS) and drying naturally (Bai et al., 2018), the plaques formed by tachyzoites were examined by microscopy.

### Anti-*T. gondii* Activity of *Pa*EO Evaluated by an Intracellular Growth Assay

A total of 10^4^ freshly released tachyzoites of the GFP-RH strain were added to HFF monolayers in 6-well plates, in DMEM with 2% FBS. Cells were incubated at 37°C in an atmosphere containing 5% CO_2_, for 4 h. The medium containing extracellular parasites was removed and fresh medium containing either *Pa*EO (3.55, 1.77, 0.89, 0.44, 0.22 mg/mL), vehicle control, or SMZ (positive control) was added to each well. After 32 h, the growth of GFP-RH was observed and photographed under a fluorescence microscope. Growth of GFP-RH was calculated using Image-Pro-Express.

### Effect of *Pa*EO on Cell Invasion by *T. gondii*

Two-color invasion experiments were performed as described by Augusto et al. (2018). A 6-well plate of HFF cells was prepared, and 3 mL of 2% FBS in DMEM was added to each well. Then, 10^4^ RH and 3.55 mg/mL *Pa*EO were added simultaneously to the wells, allowing the tachyzoites to invade host cells for 20, 40, or 60 min. The supernatant was gently removed, fixed with 2 mL methanol for 10 min, washed three times with PBS, added to 300 μL of a 5% solution of bovine serum albumin in PBS (BSA/PBS), blocked for 1 h, and washed three times with PBS. Mouse anti-*Toxoplasma* SAG1 monoclonal antibodies (mAb), diluted (1:1,000) with a 1% BSA/PBS solution, were added to each well, and incubated at room temperature for 2 h. Then, goat anti-mouse IgG H&L(FITC) secondary antibodies, diluted (1:1,000) in 1% BSA/PBS, were added to 6-well plates and incubated at room temperature for 2 h. After washing thrice with PBS, 300 μL of 0.2% Triton X-100 was added, and the mixture was left for 30 min. Cells were then gently washed three times with PBS, and 300 μL of a 5% BSA/PBS solution was added dropwise for a second blocking. The antibodies were added as per the procedure described earlier, this time using goat anti-mouse IgG H&L (Alexa Fluor^®^ 568) (ab175473) instead of the goat anti-mouse IgG H&L(FITC). Finally, 300 μL of 30% glycerol was added to each well. Five visual fields were randomly selected for observation under the 40× objective of the fluorescence microscope and the parasites in each field were counted. Three repetitions were performed to increase the accuracy of the experiment.

Tachyzoites that were unable to successfully invade the cells were dyed green by goat anti-mouse IgG H&L (FITC), while all tachyzoites in the field of vision (including the non-invading and successfully invading ones) were stained red by goat anti-mouse IgG H&L (Alexa Fluor^®^ 568) (ab175473). The difference between the tachyzoites of the two colors is termed as the absolute invasion number of tachyzoites. The ratio of the invasion number to the total number of tachyzoites is termed as the invasion rate of tachyzoites.

### Assessment of Tachyzoite Ultrastructure Using Scanning Electron Microscopy

To determine differences in the ultrastructure of tachyzoites after treatment, 1,000 purified tachyzoites were treated with *Pa*EO, and incubated at 37°C for 8 h. They were then washed twice with PBS, and fixed overnight, using 2.5% glutaraldehyde at room temperature. Gradient dehydration was carried out, using different concentrations of ethanol. The tachyzoites were coated with gold (20–30 nm) and observed by scanning electron microscopy.

### Statistical Analysis

All data were analyzed using GraphPad Prism 8.0. The anti-parasitic activity of the EOs was analyzed using an unpaired *t*-test, while the cell invasion data were processed using multiple *t*-tests, to compare the results of the test groups and those of the control group (^∗^*P* < 0.05, ^∗∗^*P* < 0.01, ^∗∗∗^*P* < 0.001).

## Results

### Cytotoxicity of EOs

**FIGURE 1:**
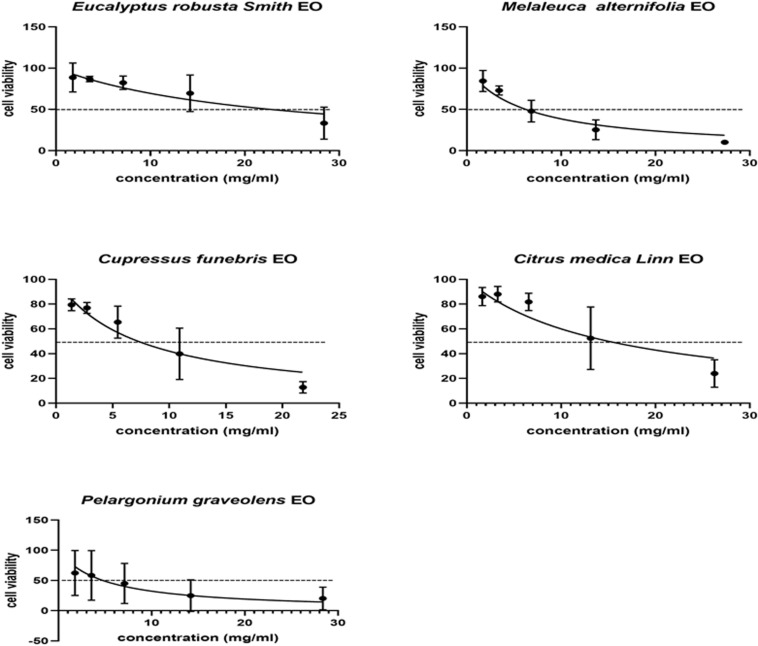
Cytotoxicity assay of five Eos. Cytotoxicity of five essential oils on HFF cells. HFF cells were treated with different concentrations of five essential oils for 24 h, respectively. Cytotoxicity was evaluated using a CellTiter 96^®^ AQueous One Solution Cell Proliferation Assay. All data are presented as with error bars and the experiments were performed in triplicate.

It was necessary to evaluate the cytotoxic potential of each EO on the same cell line as that intended to be used in the subsequent assays. The concentration that induced 50% HFF cell mortality (CC_50_) was determined using an MTT assay, and analyzed using GraphPad Prism 8.0. The CC_50_ values of *Eg*EO, *Cs*EO, *Ca*EO, *Ma*EO, *and Pa*EO were found to be 22.74, 7.25, 15.01, 6.26, and 4.77 mg/mL, respectively (Table 1 and Figure 1).

**TABLE 1:** Cytotoxic effects of five EOs.

**EOs**	**CC_5__0_ (mg/mL) (95% confidence intervals)**
*Citrus aurantifolia*	15.01 (10.28–22.40)
*Cupressus sempervirens*	7.25 (5.26–10.01)
*Eucalyptus Globulus*	22.74 (14.82–36.46)
*Melaleuca alternifolia*	6.26 (4.721–8.282)
*Pelargonium X. Asperum*	4.77 (2.075–10.47)

**Results are presented as the mean CC_50_ (cytotoxicity concentration 50%) values obtained from three independent experiments.**

### *In vitro* Anti-parasitic Activity of EOs

**FIGURE 2:**
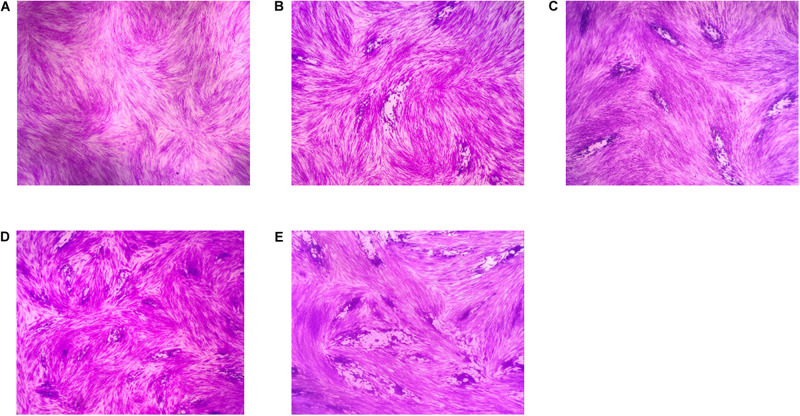
Anti-*T. gondii* activity of EOs evaluated by plaque assay. Images of *T. gondii* plaque under different concentration of *Pelargonium graveolens* EO. **(A)** HFF cells were not infected and treated. **(B)** HFF cells were infected by *T. gondii* and treated with 1.77 mg/mL *Pa*EO. **(C)** HFF cells were infected by *T. gondii* and treated with 0.44 mg/mL *Pa*EO. **(D)** HFF cells were infected by *T. gondii* and untreated. **(E)** HFF cells were infected by *T. gondii* and treated with DMSO.

**FIGURE 3:**
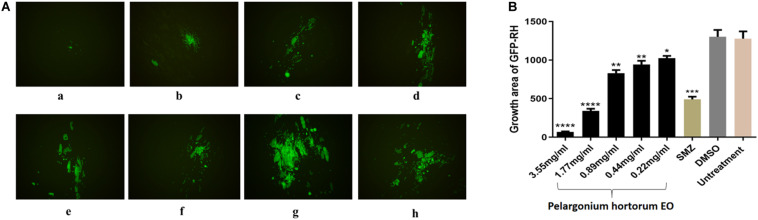
Anti-*T. gondii* activity of *Pa*EO evaluated by intracellular growth assay. **(A)** Images of growth area of *T. gondii* treated with different concentrations of *Pa*EO *in vitro.* Fluorescence area shows the growth of *T. gondii* treated with different concentrations of *Pa*EO. **(a–e)** Different concentrations of *Pa*EO, **(a)** 3.55 mg/mL; **(b)** 1.77 mg/mL; **(c)** 0.89 mg/mL; **(d)** 0.44 mg/mL; **(e)** 0.22 mg/mL; **(f)** SMZ; **(g)** DMSO; **(h)** no treatment. **(B)** The inhibition of *Pa*EO on *T. gondii* infections. The inhibition of infection for RH-GFP was measured by fluorescence area. Each bar represents the mean ± *SD* of three wells per group. *P < 0.05, **P < 0.01, ***P < 0.001, ****P < 0.0001 compared with untreated group.

A plaque test was used to screen the anti-*T. gondii* activity of the five EOs. As seen in Figure 2, we found that the plaques visible were fewer in number and smaller in size after treatment with two different concentrations of *Pa*EO, as compared to those in the DMSO-treated and untreated groups. However, no significant reduction in plaque number or size was found in the samples treated with *Er*EO, *Cf*EO, *Cm*EO, and *Ma*EO (data not shown). These results indicate that *Pa*EO is able to inhibit the growth of RH within safe concentrations, while the other four EOs exhibit no direct anti-parasitic activity. To evaluate the effect of *Pa*EO concentration on anti-parasitic activity, five different concentrations were compared using an *in vitro* inhibition assay. We found that the growth of *T. gondii* was inhibited by each of the concentrations of *Pa*EO tested (Figure 3A), and the activity increased in a dose-dependent manner (Figure 3B). The results show that the growth of *T. gondii* was significantly reduced after treatment with 3.55 and 1.77 mg/mL of *Pa*EO (68.17 vs. 1,278; 342.3 vs. 1,278, *P* < 0.0001), as compared to the untreated and 1.56% DMSO-treated groups. For the groups treated with 0.89 and 0.44 mg/mL *Pa*EO, the differences were also significant (830.3 vs. 1,278, 942.8 vs. 1,278, *P* < 0.01). The inhibition of *T. gondii* was much more significant in the groups treated with 3.55 and 1.77 mg/mL *Pa*EO, than in those treated with SMZ (68.17 vs. 490.1, 342.3 vs. 490.1, *P* < 0.01). The IC_50_ of *Pa*EO was found to be 1.426 mg/mL.

### Effect of *Pa*EO on Cell Invasion by *T. gondii*

**FIGURE 4:**
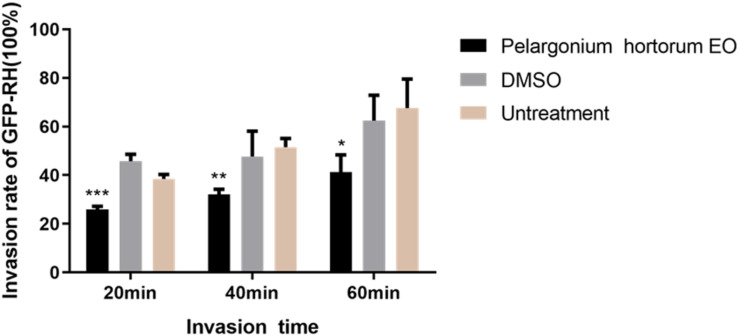
Effect of *Pa*EO on the invasion of *T. gondii.* The percentage of tachyzoites invading cells after treated with *Pa*EO for 20, 40, 60 min, respectively. Significant differences between the treated groups and untreated group are indicated. *P < 0.05, **P < 0.01, ***P < 0.001 compared with untreated group.

As summarized in Figure 4, in the 3.55 mg/mL *Pa*EO treatment group, the *T. gondii* invasion rates at 20, 40, and 60 min post-infection were found to be 25.98, 32.01, and 41.18%, respectively. In the untreated groups, invasion rates were found to be 38.50, 51.51, and 67.64%, respectively, at the three time points. Compared to the untreated group, *Pa*EO significantly reduced the invasion of *T. gondii*, especially after treatment for 60 min (*P* < 0.001). The inhibitory effect was observed to increase as the treatment time increased. No change in the invasion rate of *T. gondii* was observed in any group treated with DMSO, across all experiments.

### Tachyzoite Ultrastructure Analysis

**FIGURE 5:**
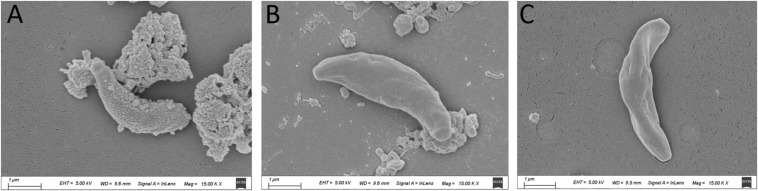
Scanning electron microscopy assay. The cells were treated with 1.77 mg/mL *Pa*EO **(A)**, DMSO **(C)**, or untreated **(B)**. After treated by *Pa*EO, the tachyzoites in the treated group shrunk and the membrane surface became rough comparing to those untreated tachtzoites, Scale bars: 1 μm.

The SEM results showed that the surface of the tachyzoites became rough after treatment with *Pa*EO. This was markedly different from the observations made in the untreated group (Figure 5). The tachyzoites also appeared smaller and shrunken after treatment with *Pa*EO, as compared to those in the untreated and DMSO-treated groups.

## Discussion

Due to the limitations of the drugs currently available for the treatment of toxoplasmosis, and the lack of drugs for chronic infections, the search for safer and more effective anti-*T. gondii* drugs is extremely important. Studying natural plant extracts is a good starting point for the development of new anti-parasitic drugs. In this study, the anti-*T. gondii* activity of five EOs was screened and evaluated *in vitro*. Among these EOs, *Pa*EO (IC_50_ = 1.426 mg/mL) was found to be the most potent inhibitor of *T. gondii* tachyzoite growth. The other four EOs did not exhibit any activity against *T. gondii*.

To determine the mechanism by which *Pa*EO inhibits the growth of *T. gondii*, invasion experiments were carried out. On treatment with *Pa*EO, the invasion rate of tachyzoites was found to decrease over time. It was therefore inferred that *Pa*EO may be able to inhibit the proliferation of tachyzoites by restricting their ability to invade host cells. *Pa*EO was able to reduce the number of plaques in the plaque assay, by inhibiting the invasion of host cells by *T. gondii*. Many plants of the genus *Pelargonium* show therapeutic effects against respiratory infections, fever, dysentery, and wounds (Ranger et al., 2011). Previous studies have shown that they have the ability to control the growth of *Spodoptera littoralis* (Farag et al., 2012), and the lone star tick, *Amblyomma americanum* (Tabanca et al., 2013). As summarized in Supplementary Material 1, linalool and geraniol are unique chemical constituents found in *Pa*EO. Zhang et al. predicted, through chemical-gene interaction analysis, that geraniol has 38 target genes, which are closely linked to a series of physiological activities (Zhang et al., 2019). DsbA, a virulence regulator, required by *Shigella sonnei* for survival, can be competitively inhibited by geraniol (Mirza et al., 2018). It has been reported that when the calcium-dependent protein kinase 1 (CDPK1) gene of *T. gondii* is suppressed, the gliding and motility of tachyzoites are affected (Johnson et al., 2012). Interestingly, linalool has also been shown to be an effective anesthetic in *Hydra* (Goel et al., 2019). Therefore, we speculate that *Pa*EO may be able to target the exercise-related genes of *T. gondii*. In addition, the anesthetic activity is consistent with that of synthetic L-quisqualic acid, which is considered to be an agonist of excitatory amino acid receptors. It was speculated that Japanese beetles may be paralyzed due to excessive muscle excitement, caused by the L-quisqualic acid in zonal geranium (Ranger et al., 2011). Zonal geranium and *Pa* belong to the same geranium family. As a protozoan, *T. gondii* does not have neuromuscular junctions like insects do. However, *Pa*EO may act instead by inhibiting a target essential for the movement of *T. gondii* tachyzoites and thereby, inhibit the invasion of cells by *T. gondii*.

The results of the SEM experiment show that after treatment with *Pa*EO, the tachyzoites became significantly smaller and contracted, and their cell membrane became rough. EOs are a mixture of many components, and can destroy the integrity of biofilms (Gucwa et al., 2018). Essid et al. (2017) speculated that *Pa*EO may be able to interfere with membrane permeability in *Candida* strains, by inhibiting the formation of long chain fatty acids (especially oleic acid), which aid cell membrane penetration, and effectively allow the parasite to carry out its biological activity (Ben Hsouna and Hamdi, 2012; Essid et al., 2017). After treatment with geranium tuber extract, the capsules of *Cryptococcus neoformans* were found to have become thinner, and the surface of *C. neoformans* was found to have shrunk, when evaluated by electron microscopy. This shrinking may be a result of dehydration, caused by the high phenol content (Samie et al., 2019). Phenol is also an important component of *Pa*EO, and may affect the membrane surface of *T. gondii*, thereby inhibiting the parasite’s movement and ability to invade cells. The electron microscopy results clearly showed that *Pa*EO damaged the cell membrane of the tachyzoites and caused them to undergo atrophy. We predict that this damage may be related to the movement of tachyzoites, which in turn affected the invasion of HFF cells, and thereby reduced the number of plaques. However, the specific mechanism is still unclear, and more in-depth research is required to gain a better understanding.

## Conclusion

In conclusion, natural extracts are a promising source for the discovery of new drugs. Our research showed that *Pa*EO can inhibit *T. gondii* at a safe concentration. This inhibitory effect may be due to the destruction of cell membranes of *T. gondii* by *Pa*EO and the resulting effect on its mobility. However, the target protein and mechanism of action of *Pa*EO on *T. gondii* are still unclear and warrant further studies.

## Data Availability Statement

The original contributions presented in the study are included in the article/Supplementary Material, further inquiries can be directed to the corresponding author/s.

## Author Contributions

S-YH and NY conceived and designed the study. NY, J-KH, MP, Z-FH, and Y-MF performed the laboratory analyses. AD and J-PT analyzed the data. S-YH drafted the first version of the manuscript. All authors critically appraised and interpreted the results and provided feedback on the manuscript, and read and approved the final version.

## Conflict of Interest

The authors declare that the research was conducted in the absence of any commercial or financial relationships that could be construed as a potential conflict of interest.
