# Supplementary material for: In vitro Anti-parasitic Activity of Pelargonium X. asperum Essential Oil Against Toxoplasma gondii
Source: Front Cell Dev Biol. 2021 Feb 18;9:616340. doi: 10.3389/fcell.2021.616340 (PMC7930326; doi:10.3389/fcell.2021.616340)
Supplement: Supplementary file 1 [file Table_1.DOCX]

**Supplementary material 1:** **Composition of essential oils**

**A :** *Pelargonium X. Asperum* EO

| **No.** | **Compound** | **%** |
| --- | --- | --- |
| 1 | *alpha*-pinene | 0.460 |
| 2 | *beta*-pinene | 0.050 |
| 3 | *beta*-myrcene | 0.280 |
| 4 | limonene | 0.170 |
| 5 | 1,8-cineole | 0.110 |
| 6 | *cis*-*beta*-ocimene | 0.150 |
| 7 | *trans*-*beta*-ocimene | 0.150 |
| 8 | *gamma*-terpinene | 0.03 |
| 9 | terpinolene | 0.04 |
| 10 | *alpha*-terpineol | 0.55 |
| 11 | para-cymene | 0.09 |
| 12 | methyl heptenone | 0.08 |
| 13 | *cis*-oxyde de rose | 1.17 |
| 14 | *trans*-oxyde de rose | 0.460 |
| 15 | menthone | 0.290 |
| 16 | *delta*-elemene | 0.190 |
| 17 | *beta*-elemene | 0.250 |
| 18 | citronellal | 0.090 |
| 19 | *iso*-menthone | 8.280 |
| 20 | *alpha*-copaene | 0.350 |
| 21 | *beta*- bourbonene | 1.100 |
| 22 | linalol | 6.460 |
| 23 | *beta*-caryophyllene | 1.550 |
| 24 | *alpha*-caryophyllene | 0.220 |
| 25 | guaia-6,9-diene | 6.760 |
| 26 | formate de citronellyle | 12.060 |
| 27 | neral | 1.000 |
| 28 | germacrene d | 0.950 |
| 29 | formate de geranyle | 6.820 |
| 30 | acetate de geranyle | 0.420 |
| 31 | *beta*-selinene | 0.420 |
| 32 | *alpha*-selinene | 0.080 |
| 33 | geranial | 0.590 |
| 34 | *trans*-piperitol | 0.870 |
| 35 | citronellol | 22.440 |
| 36 | nerol | 0.760 |
| 37 | propionate de geranyle | 1.150 |
| 38 | butyrate de geranyle | 0.880 |
| 39 | tiglate de geranyle | 0.890 |
| 40 | geraniol | 13.760 |
| 41 | oxyde de caryophyllene | 0.220 |
| 42 | acide geranique | 0.060 |
| 43 | tiglate de phenylethyle | 0.310 |
|  | total | 93.010 |

**B :** *Citrus aurantifolia* EO

| **No.** | **Compound** | **%** |
| --- | --- | --- |
| 1 | *alpha*-thujene | 0.59 |
| 2 | *alpha*-pinene | 2.217 |
| 3 | camphene | 0.062 |
| 4 | sabinene | 1.964 |
| 5 | *beta*-pinene | 11.948 |
| 6 | myrcene | 1.463 |
| 7 | *alpha*-phellandrene | 0.049 |
| 8 | *alpha*-terpinene | 0.305 |
| 9 | para-cymene | 0.118 |
| 10 | limonene | 58.257 |
| 11 | *beta*-phellandrene | 0.517 |
| 12 | (e)-*beta-*ocimene | 0.087 |
| 13 | *gamma*-terpinene | 13.797 |
| 14 | terpinolene | 0.604 |
| 15 | linalol | 0.127 |
| 16 | citronellal | 0.032 |
| 17 | terpinene-4-ol | 0.099 |
| 18 | *alpha*-terpineol | 0.216 |
| 19 | decanal | 0.069 |
| 20 | neral | 0.927 |
| 21 | geranial | 1.378 |
| 22 | *delta*-elemene | 0.132 |
| 23 | Acetate de neryle | 0.711 |
| 24 | Acetate de geranyle | 0.167 |
| 25 | *beta*-elemene | 0.122 |
| 26 | *alpha*-*cis*-bergamotene | 0.069 |
| 27 | *alpha*-*tran*s-bergamotene | 1.025 |
| 28 | *beta*-caryophyllene | 0.472 |
| 29 | *gamma*-elemene | 0.057 |
| 30 | (z)-*beta*-farnesene | 0.09 |
| 31 | *alpha*-humulene | 0.08 |
| 32 | germacrene d | 0.112 |
| 33 | alpha-bisabolene | 0.079 |
| 34 | (e,e)-*alpha*-farnesene | 0.278 |
| 34 | *beta*-bisabolene | 1.539 |
|  | total | 99.759 |

**C:** *Melaleuca alternifolia* EO

| **No.** | **Compound** | **%** |
| --- | --- | --- |
| 1 | *alpha*-thujene | 0.816 |
| 2 | *alpha*-pinene | 2.376 |
| 3 | camphene | 0.01 |
| 4 | sabinene | 0.06 |
| 5 | *beta*-pinene | 0.726 |
| 6 | *beta*-myrcene | 0.861 |
| 7 | para-mentha-1(7)8-diene | 0.004 |
| 8 | *alpha*-phellandrene | 0.342 |
| 9 | *alpha*-terpinene | 10.493 |
| 10 | para-cymene | 1.8 |
| 11 | limonene | 1.072 |
| 12 | *beta*-phellandrene | 0.877 |
| 13 | 1,8-cineole(eucalyptol) | 3.656 |
| 14 | (z)-*beta*-ocimene | 0.008 |
| 15 | *gamma*-terpinene | 22.517 |
| 16 | *cis*-hydrate de sabinene | 0.004 |
| 17 | terpinolene | 3.702 |
| 18 | linalol | 0.038 |
| 19 | *trans*-hydrate de sabinene | 0.021 |
| 20 | *cis*-para-menth-2-en-1-ol | 0.187 |
| 21 | *trans*-para-menth-2-en-1-ol | 0.124 |
| 22 | terpinene-4-ol | 41.06 |
| 23 | *alpha*-terpineol | 2.42 |
| 24 | bicycloelemene | 0.058 |
| 25 | *beta*-elemene | 0.014 |
| 26 | *alpha*-cubebene | 0.043 |
| 27 | isoledene | 0.051 |
| 28 | *alpha*-copaene | 0.124 |
| 29 | *alpha*-gurjunene | 0.285 |
| 30 | *beta*-caryophyllene | 0.343 |
| 31 | *alpha*-caryophyllene | 0.081 |
| 32 | aromadendrene | 0.914 |
| 33 | allo-aromadendrene | 0.371 |
| 34 | *alpha*-guaiene | 0.106 |
| 35 | *cis*-muurola-3.5-diene | 0.113 |
| 36 | *gamma*-muurolene | 0.071 |
| 37 | *trans*-muurola-4(14),5-diene | 0.056 |
| 38 | *trans*-cadina-1,4-diene | 0.123 |
| 39 | *gamma*-cadinene | 0.012 |
| 40 | *delta*-cadinene | 0.784 |
| 41 | *trans*-cadina-1,(6)4-diene | 0.242 |
| 42 | *delta*-selinene | 0.085 |
| 43 | viridiflorene | 0.728 |
| 44 | *cis*-calamenene | 0.274 |
| 45 | palustrol | 0.051 |
| 46 | bicyclogermacrene | 0.545 |
| 47 | spathulenol | 0.044 |
| 48 | oxyde de caryophyllene | 0.02 |
| 49 | viridiflorol | 0.201 |
| 50 | globulol | 0.197 |
| 51 | rosifoliol | 0.055 |
| 52 | 10-epi-gamma-eudesmol | 0.091 |
| 53 | 1-epi-cubenol | 0.149 |
| 54 | 1,10-di-epi-cubenol | 0.072 |
| 55 | *alpha*-muurolol | 0.027 |
|  | total | 99.504 |

**D:** *Cupressus sempervirens* EO

| **No.** | **Compound** | **%** |
| --- | --- | --- |
| 1 | tricyclene | 0.34 |
| 2 | *alpha*-pinene | 54.84 |
| 3 | *alpha*-thujene | 0.47 |
| 4 | *alpha*-fenchene | 0.46 |
| 5 | camphene | 0.35 |
| 6 | *beta*-pinene | 1.25 |
| 7 | sabinene | 0.75 |
| 8 | verbenene | 0.04 |
| 9 | *delta*-3-carene | 12.38 |
| 10 | *beta*-myrcene | 2.11 |
| 11 | *alpha*-phellandrene | 0.04 |
| 12 | *alpha*-terpinene | 0.21 |
| 13 | isosylvestrene | 0.1 |
| 14 | limonene | 2.46 |
| 15 | *beta*-phellandrene | 0.26 |
| 16 | *gamma*-terpinene | 0.3 |
| 17 | *trans*-*beta*-ocimene | 0.08 |
| 18 | meta-cymene | 0.02 |
| 19 | para-cymene | 0.29 |
| 20 | para-cymenene | 0.14 |
| 21 | meta-cymene-8-ol | 0.01 |
| 22 | para-cymene-8-ol | 0.03 |
| 23 | isoterpinolene | 0.08 |
| 24 | terpinolene | 2.14 |
| 25 | *alpha*-cubebene | 0.48 |
| 26 | *alpha*-ylangene | 0.03 |
| 27 | *alpha*-copaene | 0.15 |
| 28 | camphre | 0.11 |
| 29 | karahanaenone | 0.03 |
| 30 | linalol | 0.02 |
| 31 | *epsilon*-cadinene | 0.65 |
| 32 | *cadina*-3,5-diene | 0.16 |
| 33 | *delta*-cadinene | 0.66 |
| 34 | gamma-cadinene | 0.21 |
| 35 | cadina-1,4-diene | 0.05 |
| 36 | *alpha*-cadinol | 0.03 |
| 37 | acetate de bornyle | 1.21 |
| 38 | *beta*-elemene | 0.2 |
| 39 | *beta*-caryophyllene | 0.9 |
| 40 | *alpha*-caryophyllene | 0.4 |
| 41 | oxyde de caryophyllene | 0.01 |
| 42 | ether de methylcarvacrol | 7.41 |
| 43 | carvacrol | 0.06 |
| 44 | terpinene-4-ol | 0.34 |
| 45 | acetate de terpinen-4-yle | 0.38 |
| 46 | *cis*-thujopsene | 0.06 |
| 47 | umbellulone | 0.05 |
| 48 | *trans*-pinocarveol | 0.05 |
| 49 | *cis*-muurola-3.5-diene | 0.25 |
| 50 | *gamma*-muurolene | 0.42 |
| 51 | *alpha*-muurolene | 0.26 |
| 52 | formiate de terpinyle | 0.11 |
| 53 | acetate de terpinyle | 0.71 |
| 54 | *alpha*-terpineol | 0.11 |
| 55 | Germacrene d | 2.18 |
| 56 | *beta*-selinene | 0.21 |
| 57 | *trans*-calamenene | 0.03 |
| 58 | *cis*-calamenene | 0.03 |
| 59 | Isovalerate de decadienyle | 0.010 |
| 60 | cedrol | 0.76 |
| 61 | isopimaradiene | 0.08 |
| 62 | oxyde de manoyle | 0.04 |
| 63 | tetra-triacontane | 0.28 |
| 64 | total | 98.28 |

**E:** *Eucalyptus Globulus* EO

| **No.** | **Compound** | **%** |
| --- | --- | --- |
| 1 | isovaleraldehyde | 0.06 |
| 2 | *alpha*-pinene | 7.27 |
| 3 | camphene | 0.03 |
| 4 | *beta*-pinene | 0.23 |
| 5 | pinadiene | 0.03 |
| 6 | *beta-*myrcene | 0.36 |
| 7 | *alpha*-phellandrene | 0.7 |
| 8 | *alpha*-terpinene | 0.16 |
| 9 | *gamma*-terpinene | 2.51 |
| 10 | terpinolene | 0.04 |
| 11 | terpinene-4-ol | 0.04 |
| 12 | *delta*-terpineol | 0.03 |
| 13 | *alpha*-terpineol | 0.15 |
| 14 | limonene | 6.98 |
| 15 | 1,8-cineole(eucalyptol) | 74.85 |
| 16 | *cis*-*beta*-ocimene | 0.12 |
| 17 | *trans*-*bet*a-ocimene | 0.02 |
| 18 | para-cymene | 3.09 |
| 19 | pinol | 0.02 |
| 20 | *alpha*-para-dimethylstyrene | 0.04 |
| 21 | *alpha*-gurjunene | 0.05 |
| 22 | linalol | 0.02 |
| 23 | pinocarvone | 0.11 |
| 24 | beta-caryophyllene | 0.02 |
| 25 | aromadendrene | 0.98 |
| 26 | trans-pinocarveol | 0.53 |
| 27 | neral | 0.02 |
| 28 | Acetate de terpenyle | 0.3 |
| 29 | geranial | 0.02 |
| 30 | *trans*-para-mentha-2,8-dien-1-ol | 0.05 |
| 31 | *cis*-para-menth-2,8-dien-1-ol | 0.03 |
| 32 | epi-globulol | 0.04 |
| 33 | globulol | 0.11 |
| 34 | viridiflorol | 0.02 |
|  | total | 99.03 |
